# Resistance of *Dictyostelium discoideum *membranes to saponin permeabilization

**DOI:** 10.1186/1756-0500-3-120

**Published:** 2010-04-28

**Authors:** Valentina Mercanti, Pierre Cosson

**Affiliations:** 1 Dpt of Cell Physiology and Metabolism, Geneva Faculty of Medicine, Centre Médical Universitaire, 1 rue Michel Servet, CH1211 Geneva 4, Switzerland

## Abstract

**Background:**

Saponin is a mild detergent commonly used to permeabilize cells prior to immunofluorescence labeling of intracellular proteins. It has previously been used to that effect in *Dictyostelium discoideum *amoebae.

**Findings:**

We show that saponin, contrary to Triton X-100 or alcohol, permeabilizes at best incompletely membranes of *Dictyostelium*. In cells exposed to osmotic stress, almost complete resistance to saponin permeabilization was observed.

**Conclusions:**

Saponin should be used with special care to permeabilize *Dictyostelium *membranes. This unsusual property is presumably linked to the specific sterol composition of *Dictyostelium *membranes. It may also represent an adaptation of *Dictyostelium *to harsh conditions or to natural compounds encountered in its natural environment.

## Background

The localization of individual proteins in eukaryotic cells is often determined by immunofluorescence, a procedure requiring fixation and permeabilization of the cells before antibodies can be applied. Permeabilization of fixed cells is usually achieved by extracting membrane lipids with detergents like saponin or Triton X-100, or with alcohol. Mild permeabilization procedures are often preferred, in the hope of preserving better the cellular architecture. In particular, saponin (at concentrations of 0.1 to 0.5%) is a widely used detergent that extracts cholesterol from membranes. It is notably less prone than non-ionic detergents like Triton X-100 or NP-40 to extract membrane proteins from cellular membranes [1]. Since different cholesterol concentrations are found in various cellular membranes, saponin can be used at very low concentrations (below 0.02%) to selectively permeabilize only a few membrane compartments of mammalian cells (e.g. the plasma membrane but not the endoplasmic reticulum) [2].

*Dictyostelium *amoebae are widely used to study many facets of cellular biology, such as the organization and function of the endocytic pathway. We [3] and others [4-6] have used saponin permeabilization to perform immnofluorescence on fixed cells. In the course of our studies, we were brought to suspect that saponin permeabilization of *Dictyostelium *cells may be incomplete, particularly in starved cells undergoing multicellular development. In this study, we show that *Dictyostelium *cells exposed to various conditions may exhibit highly variable degrees of resistance to saponin permeabilization.

## Methods

### Cells and reagents

*Dictyostelium discoideum *DH1-10 cells [7] were grown at 21°C in HL5 medium (14.3 g l^-1 ^peptone (Oxoid LTD, Basingstoke, Hampshire, UK), 7.15 g l^-1 ^yeast extract (Brunschwig BD Difco, Basel, Switzerland), 18 g l^-1 ^maltose (Fluka, Buchs, Switzerland), 3.6 mM Na_2_HPO_4_, and 3.6 mM KH_2_PO_4_, pH 6.7).

Saponin prepared from Quillaja bark was from Sigma, paraformaldehyde was from Applichem and Triton X-100 from Fluka. Mouse monoclonal antibodies against the p80 endosomal marker (H161) were described previously [3].

### Immunofluorescence

To assess the efficacy of saponin permeabilization, *Dictyostelium *cells (5 × 10^5^) were deposited on a glass coverslip (20 × 20 mm) in fresh HL5 medium (2 ml), and allowed to attach at 21°C for 3 h, then fixed for 30 minutes in HL5 medium containing 4% paraformaldehyde. Fixed cells were rinsed two times with PBS, and permeabilized with saponin (0.5% in PBS for 15 minutes) or with Triton X-100 (0.1% in PBS for 3 minutes). Note that these concentrations are well above the critical micellar concentrations of saponin (0.1%) and Triton X-100 (0.02%). Permeabilized cells were pre-incubated for 15 minutes in PBS containing 0.2% BSA (PBS-BSA), then incubated for 1 hour in PBS-BSA containing an Alexa-488-coupled H161. Cells were washed three times in PBS-BSA, one time in PBS, and cellular lipids were extracted in methanol at -20°C for 2 minutes. Cells were then rinsed in PBS, pre-incubated for 15 minutes in PBS-BSA, and incubated for 1 hour in PBS-BSA containing H161 antibody coupled to Alexa-647. Finally, cells were washed three times with PBS-BSA, one time with PBS, and mounted in Mowiol [8]. Stained cells were observed in a LSM510 confocal microscope (Zeiss) and pictures were taken consecutively with identical settings to allow direct comparison between the signal intensities observed in various conditions within the same experiment.

To test permeabilization under starvation conditions, the same procedure was followed, except that cells were incubated for 30 minutes in starvation buffer (SB: 2 mM Na_2_HPO_4_, 14.7 mM KH_2_PO_4_, pH 6.0) just before fixation. Alternatively, an isotonic starvation buffer (SB+100 mM sorbitol) was used when indicated.

## Results and discussion

**Figure 1:**
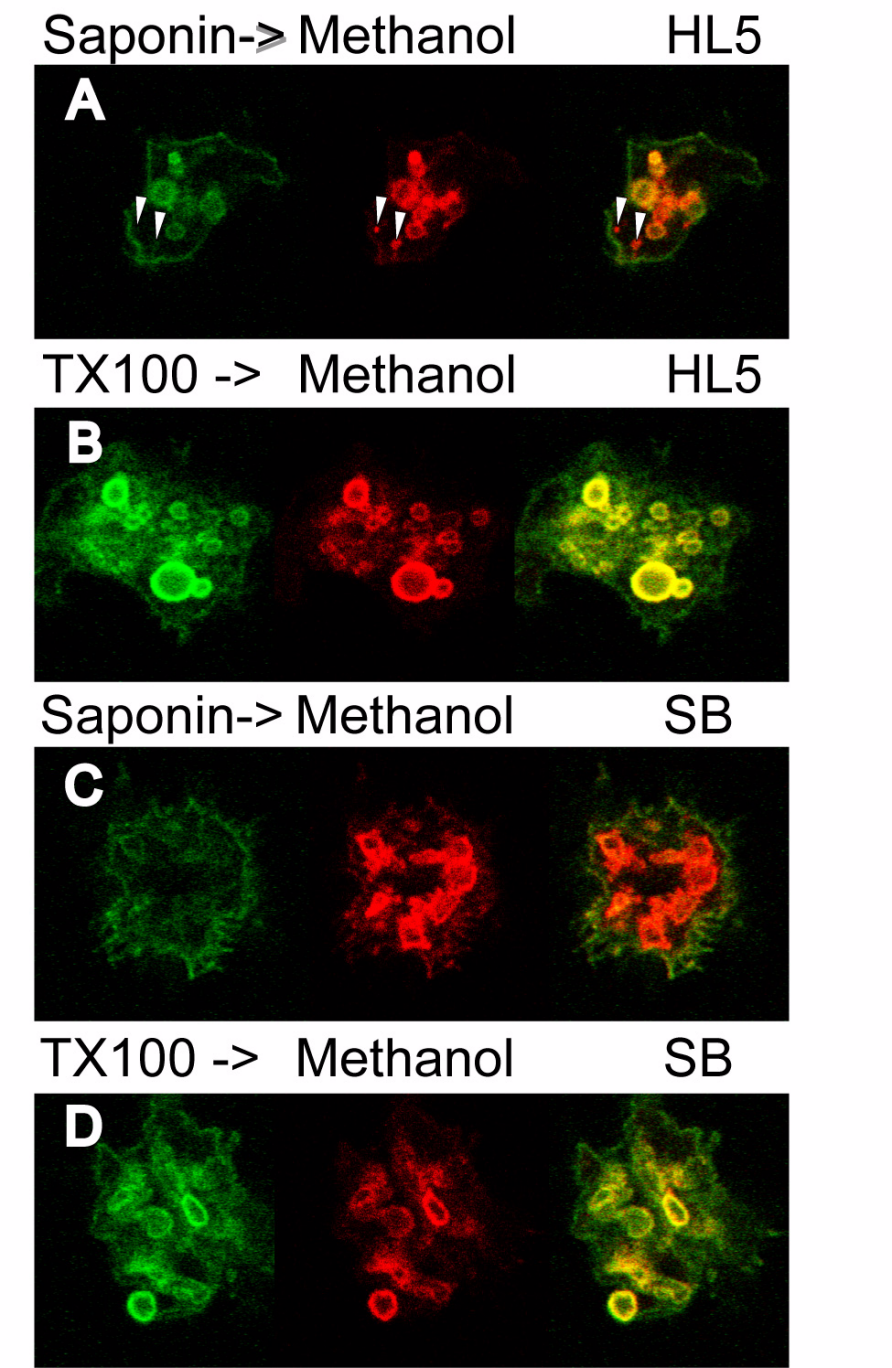
**Assessing permeabilization by detergents in *Dictyostelium *cells**. A) In order to test the permeabilization efficiency of saponin, fixed cells were permeabilized with saponin and incubated with H161 antibodies coupled to Alexa-488 (green). The cells were then further permeabilized with methanol at -20°C, and incubated with the H161 antibody coupled to Alexa-647 (red). Labeled cells were visualized in a LSM510 Zeiss confocal microscope. In cells grown in HL5 medium, the variable amounts on red and green labeling in various compartments indicated significant variations in the efficiency of saponin permeabilization. A few compartments were only stained after methanol permeabilization (arrowheads). B) Cells were treated as described in A, but saponin was replaced with Triton X-100. Triton permeabilized efficiently cellular membranes. C) In cells incubated for 30 minutes in starvation buffer (SB), virtually no intracellular staining was seen after saponin permeabilization, suggesting that membranes were highly resistant to saponin permeabilization. D) Cells exposed to SB were efficiently permeabilized with Triton X-100. All the pictures presented in this figure were from the same experiment, and taken consecutively with identical microscope settings, allowing direct comparison of labeling intensities in various conditions.

To assess directly the degree of permeabilization of *Dictyostelium *membranes with saponin, we permeabilized fixed cells with saponin (0.5%) and incubated them with an Alexa-488-coupled H161, an antibody recognizing the extracellular domain of p80, a protein present at the cell surface as well as in endosomes [3] (Figure 1A, green). The cells were then further permeabilized with methanol at -20°C, then incubated with the H161 antibody coupled to Alexa-647 (Figure 1A, red). A compartment permeabilized with saponin should be stained in yellow (red+green), while a compartment not permeabilized with saponin should be stained only in red. We observed that in cells grown in rich HL5 medium, the vast majority of endosomal compartments were labeled after saponin permeabilization (Figure 1A, green), although in every cell a few endosomes appeared to be only accessible to antibodies after permeabilization with methanol (Figure 1A, arrowheads). This result suggests that for a few endocytic compartments even at high saponin concentrations, virtually no saponin permeabilization was observed. On the contrary, in cells permeabilized with Triton X-100 instead of saponin, all compartments were stained intensely, resulting in a yellow stained merge image (Figure 1B). Direct comparison of the labeling intensity in endosomal compartments after saponin or Triton X-100 permeabilization suggests strongly that even for endosomal compartments effectively permeabilized with saponin, access of antibody was much less efficient than in compartments permeabilized with Triton X-100 (compare green fluorescence levels in Figure 1A and 1B).

**Figure 2:**
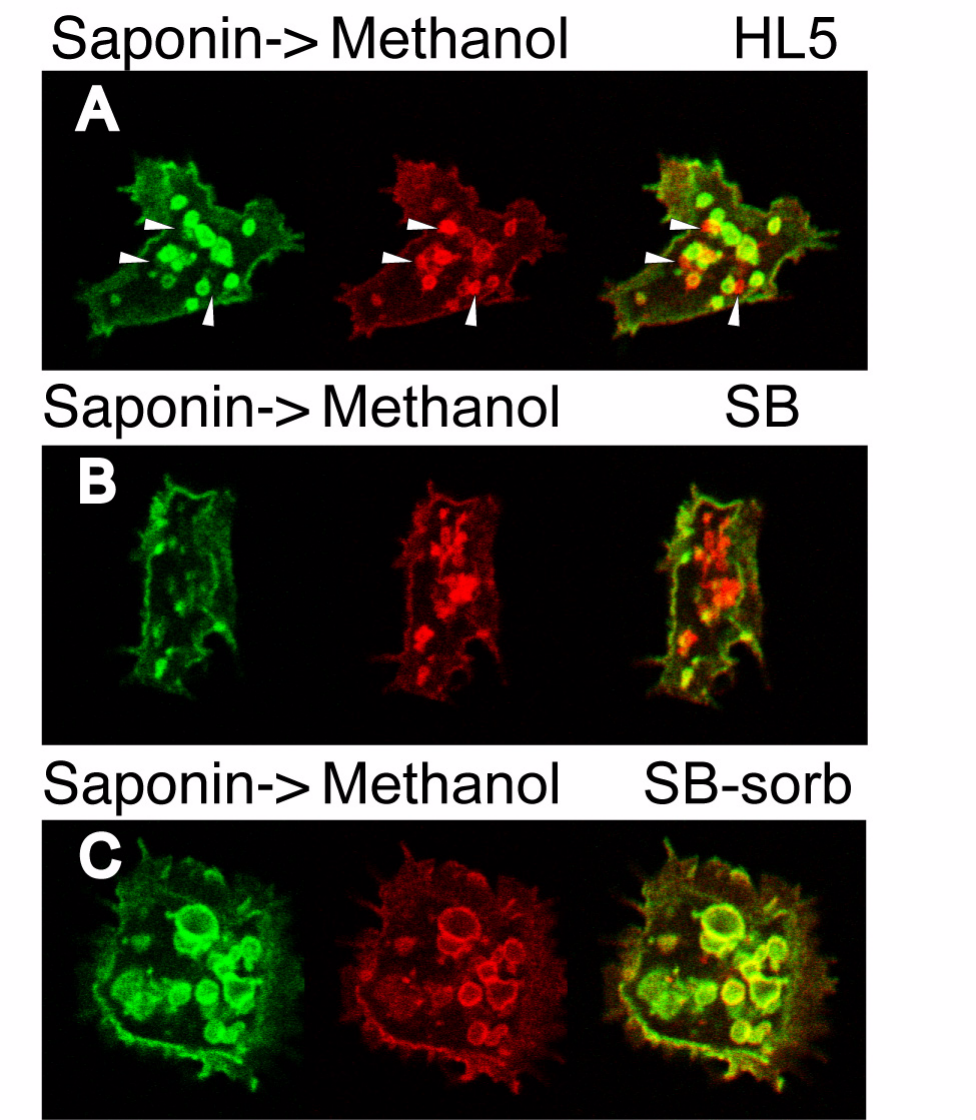
**Resistance of *Dictyostelium *membranes to saponin permeabilization is dependent on osmotic conditions**. Cells incubated in various conditions were fixed, permeabilized with saponin, stained with H161 antibody (green), then permeabilized with methanol and labeled again with H161 antibody (red) as described in the Legend to figure 1. A) Cells grown in HL5 medium. Arrowheads indicate the few compartments that were only stained after methanol permeabilization. B) Cells exposed to SB starvation buffer. C) Cells exposed to an isotonic starvation buffer (SB+100 mM sorbitol) were efficiently permeabilized with saponin suggesting that osmotic conditions, rather than starvation, modulate resistance of *Dictyostelium *membranes to saponin permeabilization. All the pictures presented in this figure were from the same experiment, and taken consecutively with identical microscope settings, allowing direct comparison of labeling intensities.

An even more striking result was obtained when cells were incubated for 30 minutes in a classical starvation buffer (SB, 17 mM phosphate buffer, pH 6.0) (Figure 1C): most endosomal compartments were not accessible to antibodies after saponin permeabilization, suggesting that saponin was incapable of efficiently permeabilizing cellular membranes in these conditions. In some cells a few compartments remained accessible to the antibody (see Figure 2B), suggesting that the plasma membrane itself is permeabilized by saponin in these conditions, while endosomal membranes exhibit resistance to permeabilization. When Triton X-100 was used instead of saponin, complete permeabilization was observed (Figure 1D).

In addition to inducing starvation, SB buffer is strongly hypotonic. In order to test if resistance to saponin permeabilization was induced by starvation or by hypoosmotic conditions, we performed the same experiment with cells exposed to an isotonic starvation buffer (SB+100 mM sorbitol). In this medium, *Dictyostelium *cells are effectively starved, since sorbitol is not metabolized [9]. Efficient saponin permeabilization was observed in these cells (Figure 2), suggesting that osmotic conditions, rather than starvation, modulate resistance of *Dictyostelium *membranes to saponin permeabilization.

Together these observations indicate that *Dictyostelium *membranes are intrinsically resistant to saponin permeabilization and that this resistance can be further enhanced by an osmotic shock. It seems likely that the resistance of *Dictyostelium *membranes to saponin reflects at least in part its specific sterol composition, that has been likened to that of plant cells [10]. It remains to be established by which mechanism osmotic conditions can modulate resistance to saponin.

From a technical point of view, partial or complete resistance of *Dictyostelium *membranes to saponin permeabilization may cause a number of artifacts in immunofluorescence experiments. It may for example lead to underestimate the amount of intracellular protein relative to surface protein (compare Figure 1A and 1B), or to believe that some proteins are relocated to the cell surface in starved *Dictyostelium *cells (compare Figure 1A and 1C). On the other hand, in combination with appropriate controls, saponin permeabilization may provide a tool to study the peculiar properties of *Dictyostelium *membranes. While this study was focused on a marker of endocytic compartments, a similar approach may establish to what extent other cellular membranes are resistant to saponin extraction.

Finally, from an ecological perspective, saponins are synthesized by a variety of plants, and have been proposed to function as antimicrobial phytoprotectants [11]. It seems plausible that *Dictyostelium*, a soil-dwelling amoeba, may be naturally exposed to plant saponins, and might benefit from being resistant to its detergent activities. Resistance of endosomal membranes to permeabilization may also limit escape of phagocytosed microorganisms from phagosomes, thereby preventing their access to the cell's cytosol.

## Competing interests

The authors declare that they have no competing interests.

## Authors' contributions

PV and VM both designed and performed experiments, and wrote the manuscript. Both authors read and approved the final manuscript.

## References

[B1] Goldenthal KL Hedman K Chen JW August JT Willingham MC Postfixation detergent treatment for immunofluorescence suppresses localization of some integral membrane proteins J Histochem Cytochem 1985 33 813 20 3894499 10.1177/33.8.3894499

[B2] Wassler M Jonasson I Persson R Fries E Differential permeabilization of membranes by saponin treatment of isolated rat hepatocytes. Release of secretory proteins Biochem J 1987 247 407 15 3426543 10.1042/bj2470407 PMC1148424

[B3] Ravanel K de Chassey B Cornillon S Benghezal M Zulianello L Gebbie L Letourneur F Cosson P Membrane sorting in the endocytic and phagocytic pathway of Dictyostelium discoideum Eur J Cell Biol 2001 80 754 64 10.1078/0171-9335-00215 11831389

[B4] Bush JM Cardelli JA Processing, transport, and secretion of the lysosomal enzyme acid phosphatase in Dictyostelium discoideum J Biol Chem 1989 264 7630 6 2651446

[B5] Zhu Q Clarke M Association of calmodulin and an unconventional myosin with the contractile vacuole complex of Dictyostelium discoideum J Cell Biol 1992 118 347 58 10.1083/jcb.118.2.347 1629238 PMC2290049

[B6] Sesaki H Wong EF Siu CH The cell adhesion molecule DdCAD-1 in Dictyostelium is targeted to the cell surface by a nonclassical transport pathway involving contractile vacuoles J Cell Biol 1997 138 939 51 10.1083/jcb.138.4.939 9265658 PMC2138044

[B7] Cornillon S Pech E Benghezal M Ravanel K Gaynor E Letourneur F Bruckert F Cosson P Phg1p is a nine-transmembrane protein superfamily member involved in dictyostelium adhesion and phagocytosis J Biol Chem 2000 275 34287 92 10.1074/jbc.M006725200 10944536

[B8] Heimer GV Taylor CE Improved mountant for immunofluorescence preparations J Clin Pathol 1974 27 254 6 10.1136/jcp.27.3.254 4598883 PMC478083

[B9] Smith EW Lima WC Charette SJ Cosson P Effect of starvation on the endocytic pathway in Dictyostelium cells Eukaryot Cell 9 387 92 10.1128/EC.00285-09 20097741 PMC2837978

[B10] Nes WD Norton RA Crumley FG Madigan SJ Katz ER Sterol phylogenesis and algal evolution Proc Natl Acad Sci USA 1990 87 7565 9 10.1073/pnas.87.19.7565 11607106 PMC54788

[B11] Papadopoulou K Melton RE Leggett M Daniels MJ Osbourn AE Compromised disease resistance in saponin-deficient plants Proc Natl Acad Sci USA 1999 96 12923 8 10.1073/pnas.96.22.12923 10536024 PMC23166

